# MiR-338-5p Inhibits EGF-Induced EMT in Pancreatic Cancer Cells by Targeting EGFR/ERK Signaling

**DOI:** 10.3389/fonc.2021.616481

**Published:** 2021-04-15

**Authors:** Jian Sun, Lin Chen, Ming Dong

**Affiliations:** Department of Gastrointestinal Surgery, The First Hospital, China Medical University, Shenyang, China

**Keywords:** miR-338-5p, epidermal growth factor (EGF) signaling, epithelial-mesenchymal transition (EMT), migration, invasion

## Abstract

The epidermal growth factor (EGF) pathway plays critical roles during cancer cell epithelial-mesenchymal transition (EMT) process and metastasis. Epidermal growth factor receptor (EGFR), as one of the important receptors of EGF, undergoes autophosphorylation with the stimulation of EGF and activates MAPK/ERK, PI3K/Akt/mTOR, and other pathways. Here, we identified EGFR was a target of miR-338-5p. Upon EGF treatment, overexpression of miR-338-5p not only downregulated EGFR expression and inhibited MAPK/ERK signaling, but also inhibited EMT and metastasis process of pancreatic cancer (PC) cells. In the clinical pathological analysis, miR-338-5p was significantly down-regulated in 44 pairs PC tissues and its expression was negatively associated with lymph node metastasis and AJCC stage. Furthermore, Overexpression of EGFR partially reversed the protective effect of miR-338-5p overexpression on EGF-mediated migration and invasion in PC cells. Taken together, miR-338-5p controls EGF-mediated EMT and metastasis in PC cells by targeting EGFR/ERK pathways. Here, we hope to provide new insights into the molecular mechanisms of pancreatic cancer, and may help facilitating development of EGFR-based therapies for human cancer.

## Introduction

Pancreatic ductal adenocarcinoma cancer (PDAC), the fourth leading cause of cancer-related death in the United States, remains one of the deadliest malignancies, with a 5-year survival rate of 9% (1). PDAC is often diagnosed at advanced stages that are locally invasive and/or widely metastatic, which precludes the possibility for potentially curative resection. In cases that are resectable, lymph node involvement is common, and cancer cell invasion or spread into peripancreatic or distant lymph nodes is associated with increased risk of disease recurrence and a poor prognosis (2). It has been confirmed that epithelial-mesenchymal transition (EMT) is a prominent process promoted aggressive local invasion and distant metastasis in pancreatic cancer (3).

The epidermal growth factor receptor (EGFR) family has been demonstrated to strongly affect EMT process in many types of cancer, including pancreatic (4–6). Moreover, elevated EGFR expression is detected during tumor progression from early pancreatic intraepithelial neoplasia to PDAC and has been recognized as the essential molecular alteration in pancreatic carcinogenesis (7). So far, in a Phase III trial, erlotinib, an EGFR tyrosine kinase inhibitor, has been proven effective in modestly increasing overall survival, in association with gemcitabine (8). However, due to the associated toxicity and to the really small improvement, the use of this combination has never found a role in clinical practice.

In this study, we examined the expression of EGFR protein and miR-338-5p in PC tissues and cell lines. found that miR-338-5p was a key negative regulator of EGFR, downregulation of miR-338-5p was associated with the JACC stage and lymph node status. Overexpression of miR-338-5p inhibited EGF-induced EMT in PC through EGFR/RAS/ERK signaling *in vitro* and vivo. To some extent, miR-338-5p has the potential to become a novel strategy in PC for EGFR-targeted therapy.

## Materials and Methods

### Cell Culture and Tissue Samples

Human PC cell lines, AsPC-1, BxPC-3, PANC-1, MIA PaCa-2, and SW1990 were obtained from the Cell Bank of the Chinese Academy of Sciences (Shanghai, China). Capan-2 cell line was purchased from the Bei Na Culture Collection (Beijing, China). PANC02 cell line was obtained from the Cell Biology Laboratory of China Medical University. All these cell lines were maintained in recommended growth media with 10% fetal calf serum (Gibco Invitrogen, Carlsbad, CA). All cell lines were maintained according to the medium recommended by ATCC, supplemented with 10% fetal calf serum (Gibco Invitrogen, Carlsbad, CA), 100 U/ml penicillin, and 100 ng/ml streptomycin (Beyotime, China). The cells were cultured at 37°C in a humidified chamber supplemented with 5% CO2.

The present study was approved by the Ethics Committee of Eastern Hospital of Hepatobiliary Surgery. All 40 paraffin-embedded and 44 fresh ductal adenocarcinoma samples were obtained from patients who underwent surgical resection at the First Hospital of China Medical University (Shenyang, China) between 2010 and 2019. All the fresh specimens were snapped-frozen and stored in liquid nitrogen.

### Immunohistochemistry

Paraffin-embedded tissues were sliced into sections of 4-um thickness, followed by dewaxing with dimethylbenzene and hydration in graded ethanol. Antigens were retrieved by high pressure method. Endogenous peroxidase was blocked using 3% hydrogen peroxide, and tissues were added with 10% normal goat serum. Then the slices were incubated with primary antibodies: EGFR (Proteintech, 1:200), followed by 4°C overnight. Subsequently, slices were incubated with the secondary antibodies, treated with streptavidin–peroxidase reagent. The incubated sections were visualized with diaminobenzidine (DAB), and then counterstained by hematoxylin, and detected under microscope. Five high-power fields (400×) were randomly selected for each section. Staining intensity was scored as 0–3 (negative, weak, medium, and strong). Extent of staining was scored as 0 (< 5%), 1 (5–25%), 2 (26–50%), 3 (51–75%), and 4 (> 75%) according to the positive staining areas to the whole carcinoma. The final scores were calculated by three pathologists. Multiplication of the two scores was the final score ranging from 0 to 12. A final score >6 was defined as a positive expression.

### Hematoxylin and Eosin Staining

In short, the slides were stained as follows: after dewaxing, hematoxylin for 5 min, 1% hydrochloric acid ethanol for 2 s, water washing for 2 min, 1% water soluble eosin for 3 min, then a series of alcohol dehydration for 30 s, xylene for 2 min.

### Cell Transfection and Virus Infection

For transient transfection, miRNA mimics, inhibitors, siRNAs (GenePharma Co, Ltd) and their negative control oligonucleotides (NC) were transfected by Lipofectamine 3000 (Invitrogen, Carlsbad, CA, USA) according to the manufacturer’s protocol. Lentivirus vector (GV492) mediated EGFR overexpression (EGFR-OE) and corresponding negative control vector (EGFR-NC) were synthesized from Genechem (Shanghai, China). PANC-1 and Capan-2 were used to construct EGFR stable expression PC cells following puromycin selection. The related sequences are shown in **Table S1**. The vector information was shown in **Figure S1**.

### Western Blot

The PC cells were seeded into 12-well plates with 1×10^5^ cells/ml with or without EGF (50 ng/ml). and cultured 48 h after transfection. Total proteins were extracted from cells or tissues with RIPA buffer (50 mM Tris pH 7.4, 150 mM NaCl, 1% Triton X-100, 1% sodium deoxycholate, 0.1% SDS) containing 1 mM PMSF and phosphatase inhibitor (Bimake, USA). The total protein concentration was determined by BCA Protein Assay Kit (TaKaRa, Japan). Proteins were separated on a 10% SDS–PAGE and then transferred onto PDVF membranes. Subsequently, membranes were blocked with 5% skimmed milk and incubated with primary antibodies: EGFR (Proteintech), the phosphorylated EGFR at tyrosine 1045 (pEGFR1045, Cell signaling technology) and at tyrosine 1068 (pEGFR1068, Abcam), E-cadherin (Abcam), c-Myc (Proteintech), ERK (Cell signaling technology), p-ERK (Cell signaling technology), MMP9 (Proteintech), mTOR (bimake), the phosphorylated mTOR at Ser2448 [p-mTOR (2448), Cell signaling technology], Akt (Proteintech), the phosphorylated Akt at Ser473 [p-Akt (ser473), Cell signaling technology], GAPDH (Proteintech). On the following day, membranes were incubated with horseradish-peroxidase-conjugated secondary antibodies (Proteintech). Protein bands were detected with an ECL detection kit (Thermol Biotech Inc, USA).

### RNA Isolation and Real-Time qRT–PCR

Total RNA was extracted from cells or tissues by using the Trizol total RNA extraction kit (TaKaRa, Japan). For the detection of miR-338-5p expression, total RNA was reverse-transcribed with a miR-338-5p specific RT primer (GenePharma, China) and microRNA Reverse Transcription Kit (GenePharma, China). amplified with cDNA amplification primers (GenePharma, China). The SYBR Green PCR kit (GenePharma, China) was used for qRT–PCR according to the manufacturer’s protocols. All following primer sequences were list in **Table S2**. The expression levels of miR-338-5p were normalized to the U6 levels.

### Epithelial–Mesenchymal Transition Construction

Capan-2 and PANC-1 cell transfected with negative control, miR-338-5p mimics, or inhibitor were treated with 50 ng/ml EGF (Peprotech, RockyHill, New Jersey, USA) or 1%BSA (Sigma) triple times within 72 h, respectively. Cells were cultured with recommended growth media containing 2% FBS to enhance the efficiency of EGF. The EMT construction was verified by the observation of EMT-like cell morphology (a spindle-shaped and fibroblast-like morphology), EMT-enhanced cell invasion and migration, and EMT-induced the change of EMT markers.

### Invasion and Migration Assay

After transfection for 48 h (pretreated with EGF for 48 h), cells were harvested and resuspended in serum-free culture medium. The upper chamber is pretreated with Matrigel (8.0 μm, BD, Biosciences). Then, 3×10^4^ cells were seeded into the transwell upper chamber (BD Biosciences, Sparks, MD, USA) with FBS-free growth media plus EGF. Medium with 10% serum was added to the lower chamber as a chemoattractant. After incubation for 16 h, the cells on the upper membrane surface were removed, and cells that had transferred to the lower surface were fixed with cold methyl alcohol for 30 min and stained for 25 min with crystal violet dye. Then, invasion cells were counted in five randomly selected fields per chamber. For cell migration assay, the procedures are the same the invasion assay, except for the Matrigel coating.

### *In Vivo* Xenograft Model

To study liver metastasis of primary tumor, a total of 12 four-week-old mice (C57B6) were used to construct liver metastasis model. PANC-02 cancer cells transfected mimics-NC or mimics-338-5p were resuspended with PBS respectively (5×10^6/ml). After the spleen was identified and exposed, 200 μl resuspension was injected into the lower middle part of splenic capsule of male C57B6 mice. A cotton swab was used to avoid bleeding and leakage from the injection site. The mice were sacrificed 4 weeks later. Take out the liver and count the number of liver metastases.

### Statistical Analysis

The results are presented as the means ± SD of at least three independent experiments. Differences between two groups were estimated with t test or one-way ANOVA. Overall survival curves were plotted according to the Kaplan–Meier method, and the log-rank test was used for comparison. To analyze the relationship between the expression of miR-338-5p and EGFR in PC tumor samples, Chi square test was used. All statistical analyses were performed using SPSS Version 21 software (Chicago, IL, USA). The differences were considered statistically significant at P <0.05.

## Results

### Epidermal Growth Factor Receptor Is Elevated at Protein Level in Pancreatic Cancer and Predicts Poor Prognosis

**Figure 1:**
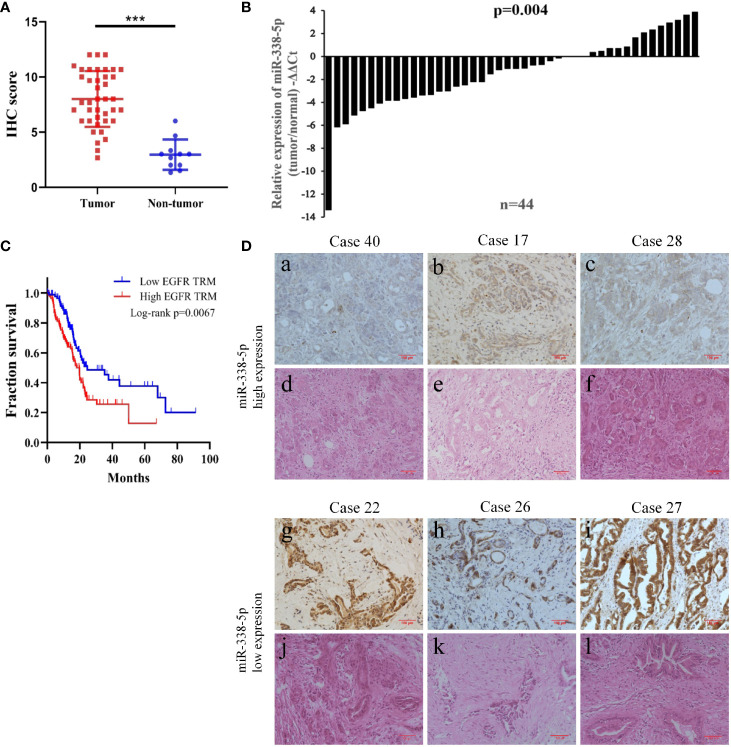
The low expression of miR-338-5p in PC tissues was negatively correlated with EGFR expression. **(A)** The expression of EGFR in the tumor tissues and normal tissues was evaluated by immunohistochemistry. **(B)** miR-338-5p levels were detected in 44 paired PC tissues by qRT-PCR. **(C)** EGFR high expression was correlated with a poor survival rate of pancreatic cancer patients (n = 174, P < 0.01). Data was analyzed using Kaplan Meier curve **(D)** The IHC results of EGFR and HE staining in corresponding PC tissues. (a–c) A low expression level of EGFR was observed in tissues with high expression levels of miR‐338-5p. (g–i) A high expression level of EGFR was observed in tissues with low expression levels of miR‐338-5p. (d–f, j–l) H&E staining results corresponding to a,–c, g–i. ***P < 0.001.

First, we assessed the expression level of EGFR by IHC in 40 pancreatic cancer tissues and 20 paired normal pancreatic tissues. IHC showed that EGFR was overexpressed in 29 cases of total 40 PC tissues (29/40, 72.5%), which was much higher than that in paired adjacent normal pancreas (8/20, 40%). As shown in **Figure 1A**, the expression of EGFR was significantly up-regulated in cancer tissues than normal pancreatic tissues (P < 0.001).

Additionally, bioinformatic analysis using the TCGA database allowed us to detect RNA sequencing data of 174 pancreatic cancer patients with complete survival statistics (**Figure 1C**). This revealed a strong correlation between poor survival and high EGFR expression in human pancreatic cancer.

### miR-338-5p Is Down-Regulated in Pancreatic Cancer Tissues and Associated With Epidermal Growth Factor Receptor Expression

In order to assess the relevance of miR-338-5p in human pancreatic cancer, we detected and compared the expression levels of miR-338-5p in 44 paired PC tissues and corresponding adjacent non-tumor tissues by qRT-PCR. As shown in **Figure 1B**, miR-338-5p expression was significantly reduced in 70% of the PC tissues (31/44). Then, according to the median of miR-338-5p level, the sample was divided into high (above median, n = 22) and low (below median, n = 22) expression group, to explore the correlation between miR-338-5p expression and clinical pathological factors in patients with PC. As shown in **Table 1**, the expression level of miR-338-5p was lower in Lymph node positive group (-5.44 to -1.21) than that in Lymph node negative group (-1.56 to 0.23) (p = 0.014). Similarly, miR-338-5p level was also lower in IIB and III group (-5.51 to -1.40) than that in I and IIA group (-1.58 to 0.27) (p = 0.012). However, no significant difference was observed about gender, age, tumor size, differentiation, and T stage.

**Table 1:** Correlations between miR-338-5p and clinicopathologic parameters in PC patients.

Parameters	No. (n = 44)	Expression of miR-338-5p		*P* value
		Mean	95% CI	
Gender				
Male	29	-1.26	-2.19 to -0.41	0.597
Female	15	-1.81	-4.09 to 0.35	
Age				
< 60	21	-1.48	-3.11 to -0.04	0.945
≥ 60	23	-1.41	-2.37 to -0.35	
Tumor size (cm)				
≤ 2	15	-1.37	-2.68 to 0.12	0.909
> 2	29	-1.49	-2.85 to -0.33	
Differentiation				
Well, moderate	30	-1.44	-2.78 to -0.25	0.995
Poor	14	-1.45	-2.64 to -0.06	
T stage				
T1+T2	27	-0.92	-1.83 to 0.00	0.178
T3+T4	17	-2.27	-4.33 to -0.49	
Lymph node status				
Negative	31	-0.69	-1.56 to 0.23	0.014^*^
Positive	13	-3.24	-5.44 to -1.21	
AJCC stage				
I+IIA	29	-0.63	-1.58 to 0.27	0.012^*^
IIB+III	15	-3.19	-5.51 to -1.40	

PC, pancreatic cancer.

The mean of the relative miR‐338-5p expression is showed as log2 (fold change) value and 95% confidence interval (CI).

AJCC stage, according to the 7th edition of American Joint Committee on Cancer.

^*^P < 0.05.

Combined with the results of immunohistochemistry, we found that EGFR was always negatively expressed in tissues highly expressed miR-338-5p, and it was always positively expressed in tissues exhibiting low miR-338-5p expression (**Figure 1D**). The results reflect the negative correlation between the expression of miR-338-5p and EGFR (**Table 2**) (P = 0.037).

**Table 2:** Association between the expression of miR-338-5p and EGFR in PC cases (N = 19).

EGFR	Expression of miR-338-5p		*P* value
	High	Low	
Positive	4	7	0.037^*^
Negative	7	1	

Fisher’s exact test was used for analysis.

^*^P < 0.05.

### Epidermal Growth Factor Receptor Is a Target of miR-338-5p

**Figure 2:**
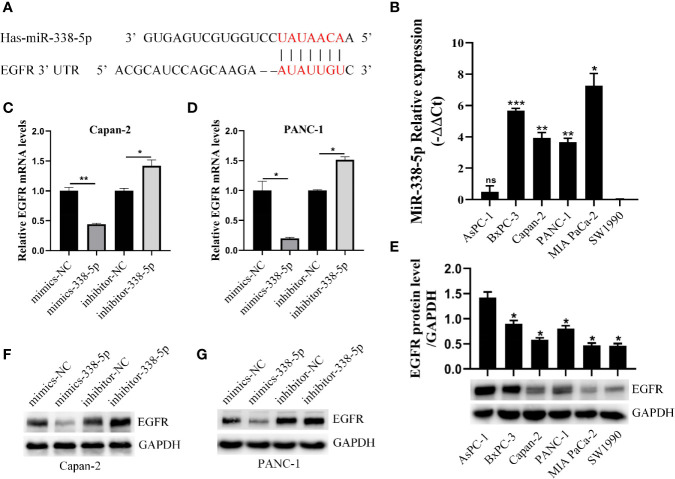
miR-338-5p downregulated the expression of EGFR at the post-transcriptional level. **(A)** The interaction between miR-338-5p and the 3’-untranslated region of EGFR was predicted by TargetScan software. **(B)** qRT-PCR analysis of miR-338-5p expression in six PC cell lines and all cell lines are calibrated with SW1990. **(C, D)** qRT-PCR analysis of EGFR mRNA expression following transfection with the miR-338-5p mimics, miR-338-5p inhibitor, and their respective normal control in Capan-2 **(C)** and Panc-1 **(D)**. **(E)** Western blot analysis of EGFR expression in six PC cell lines and all cell lines are calibrated with AsPC-1. **(F, G)** Western blot analysis of EGFR expression following transfection with the miR-338-5p mimics, miR-338-5p inhibitor, and their respective normal control in Capan-2 **(F)** and PANC-1 **(G)**. All data are shown as the mean ± SEM. *P < 0.05; **P < 0.01; ***P < 0.001.

Considering the differential expression of EGFR in pancreatic cancer and adjacent cancers, we were eager to find the epigenetic regulation mechanism of EGFR, especially the regulation effect of microRNAs on EGFR. Two databases (TargetScan and MiRDB) were used to predict the miRNAs targeting EGFR and found that potential target sites in the 3’UTR of EGFR that can interact with miR−338-5p (**Figure 2A**). Further, we examined the basal level of miR-338-5p and EGFR protein in six PC cell lines (SW1990, AsPC-1, PANC-1, Capan-2, BxPC-3, and MIA PaCa-2). Interestingly, we found differential expression of miR-338-5p in cell lines in which AsPC-1 and SW1990 (two metastatic PC cell lines) showed relatively lower level of miR-338-5p expression, whereas the other four primary PC cell lines PANC-1, Capan-2, BxPC-3, and MIA PaCa-2, showed higher expression (**Figure 2B**). The loss of expression of miR-338-5p was well-correlated with higher level of EGFR expression in four PC cell lines, with the exception of SW1990 and BxPC-3 as shown in **Figure 2E**. SW1990 cell line exhibited lower levels of both miR-338-5p and EGFR expression. In contrast, BxPC-3 cell line showed higher levels of both miR-338-5p and EGFR expression. PANC-1 and Capan-2 cell lines that appropriately expressed miR-338-5p and EGFR were used for subsequent experimental studies.

We transfected PANC-1 and Capan-2 with the miR‐338-5p mimics, miR‐338-5p inhibitor, and their corresponding NC, respectively. The transfection efficiency of miR-338-5p was detected by qRT‐PCR (**Figure S1**). As expected, the expression of EGFR protein (**Figures 2F, G**) and mRNA (**Figures 2C, D**) levels were strikingly downregulated in miR-338-5p overexpression cells, and upregulated in cells transfected with miR-338-5p inhibitor.

### miR-338-5p Inhibits Epidermal Growth Factor-Induced Epithelial–Mesenchymal Transition, Migration, and Invasion in Pancreatic Cancer Cell Lines

**Figure 3:**
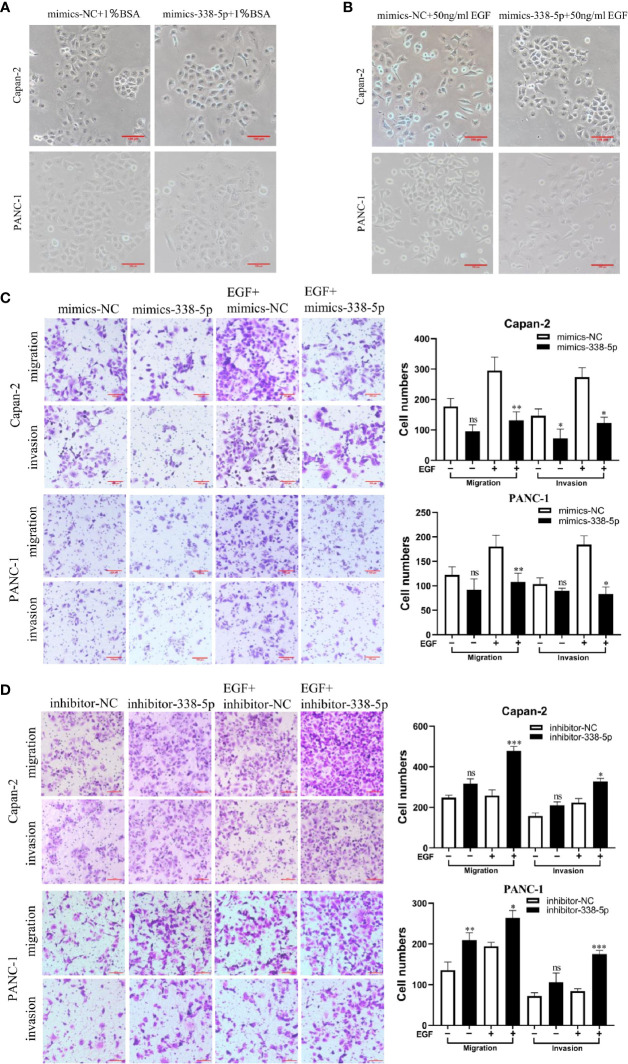
miR-338-5p promotes pancreatic cancer cells EMT, migration and invasion *in vitro*. **(A)** without EGF treatment, the Cell morphology of Capan-2 and PANC-1 cells in mimics NC, and miR-338-5p mimics groups. **(B)** Upon EGF treatment (50 ng/ml), fibroblastoid-like phenotype of Capan-2 and PANC-1 cells in mimics NC, and miR-338-5p mimics groups. **(C)** With or without EGF treatment, Capan-2 cells, and PANC-1 cells transfected with mimics NC, miR-338-5p mimics, detected by transwell migration and invasion assay. Scale bar, 100 μm. **(D)** Migration and invasion of Capan-2, and PANC-1 cells transfected with inhibitor NC, or miR-338-5p inhibitor. Scale bar, 100 μm. All data are shown as the mean ± SEM. *P < 0.05; **P < 0.01; ***P < 0.001; ns, no significance.

To determine the effect of miR‐338-5p on PC cell functions, Capan-2 and PANC-1 were transfected with miR‐338-5p mimics, or the corresponding negative control (NC), respectively. After EGF treatment, both Capan-2 and PANC-1 cells showed a cell morphology similar to EMT: the cells lost their epithelial characteristics, and presented a spindle-like and fibroblast-like morphology. Compared with the NC group, overexpression of miR-338-5p alone had no significant effect on cell morphology. However, upon EGF stimulation, Capan-2 and PANC-1 cells treated with miR-338-5p mimics recovered their original cell morphology with almost no spindle-shaped and fibroblast-like morphology (**Figures 3A, B**). Overall, miR-338-5p significantly inhibited EGF-induced EMT-like cell morphology.

As shown in the results of transwell assay, EGF significantly stimulated cell invasion and migration in both Capan-2 and PANC-1 cells. Upon EGF, a significant decrease of cell invasion and migration were found in miR-338-5p overexpression groups compared with NC groups in both cells (**Figure 3C**). Similarly, miR-338-5p silencing promoted cell invasion and migration upon EGF stimulation (**Figure 3D**). However, miR-338-5p overexpression alone without EGF treatment partially inhibited cell invasion and migration (**Figure 3C**). While miR-338-5p silencing alone without EGF partially promoted cell invasion and migration (**Figure 3D**). Namely, this movement trend is much more remarkably with EGF treatment.

**Figure 4:**
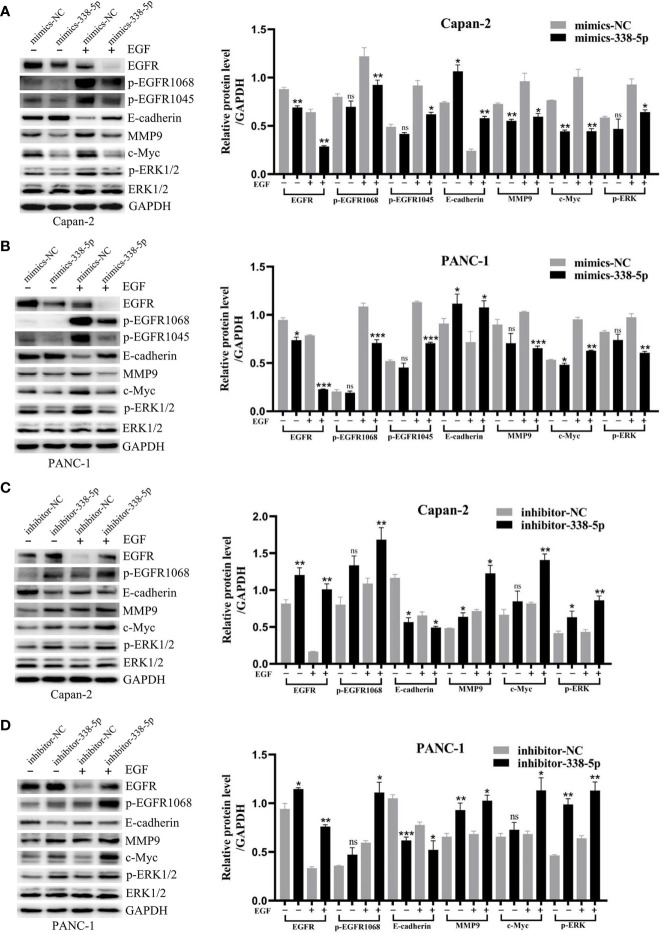
MiR-338-5p inhibited EGF-induced EMT and EGFR/ERK signaling. **(A, B)** With or without EGF treatment, the protein levels of EGFR/ERK signaling and EMT target genes in mimics NC and mimics-338-5p transfected Capan-2 **(A)**, and PANC-1 **(B)** cells detected by western blotting. **(C, D)** With or without EGF treatment, the protein levels of EGFR/ERK signaling and EMT makers in inhibitor NC and inhibitor-338-5p transfected Capan-2 **(C)**, and PANC-1 **(D)** cells detected by western blotting. All data are shown as the mean ± SEM. *P < 0.05; **P < 0.01; ***P < 0.001; ns, no significance.

To better understand the mechanism by which miR-338-5p regulates cell EMT, migration, and invasion, we examined the expression of several key proteins in EGF signal and EMT processes, including EGFR, p-EGFR, p-ERK, p-Akt, p-mTOR, E-cadherin, MMP9, and c-Myc. Western blot analysis showed that under EGF stimulation, overexpression of miR-338-5p significantly increased the expression of Epithelial marker E-cadherin and decreased the expression of MMP9, c-Myc, and p-ERK. Meanwhile, lower level of EGFR, p-EGFR1068 and p-EGFR1045 were also observed in mimics-338-5p group under EGF treatment (**Figures 4A, B**). In contrast, inhibition of miR-338-5p decreased the levels of E-cadherin and promoted the expression of EGFR, p-EGFR1068, p-EGFR1045, MMP9, c-Myc, and p-ERK (**Figures 4C, D**). However, miR-338-5p overexpression or silence alone without EGF stimulus partially changed MMP9, c-Myc, and p-ERK expression, and the changes of total ERK was not very significant for all the intervention factors (**Figures 4A–D**). Finally, compared with the control group, we did not find changes in p-Akt, Akt, p-mTOR, and mTOR caused by overexpression of miR-338-5p with or without EGF stimulation (**Figure S1**).

All these results support a role of miR-338-5p in inhibiting EGF induced EMT *in vitro*.

### MiR-338-5p Inhibited Epidermal Growth Factor-Induced Epidermal Growth Factor Receptor/ERK/MAPK Signaling in Both Cell Lines

**Figure 5:**
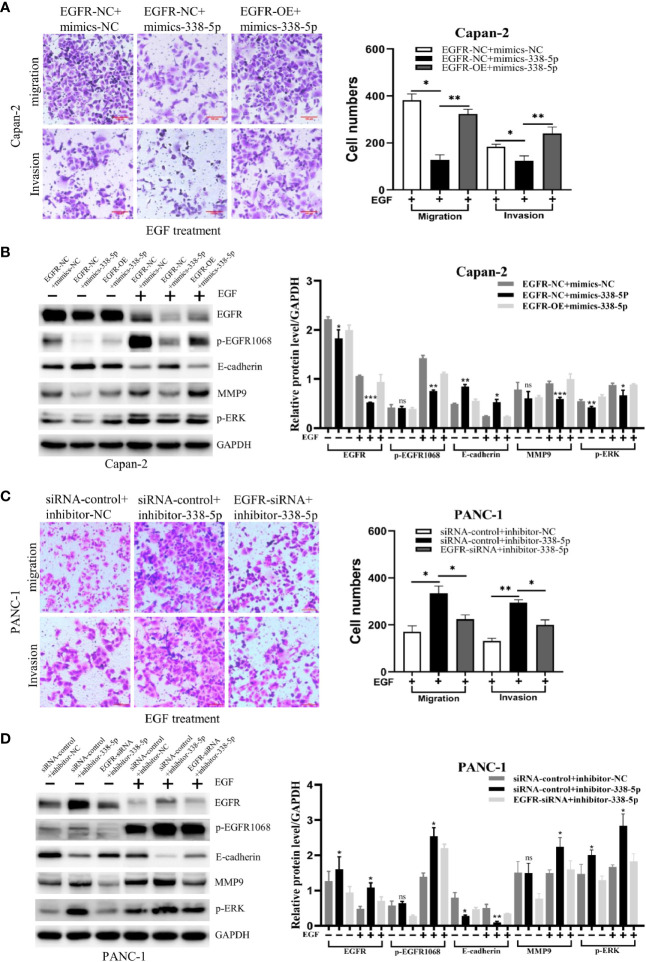
EGFR mediates the effects of miR-338-5p on pancreatic cancer cells. **(A, B)** EGFR overexpression weakened migration, invasion **(A)** and the related protein levels **(B)**, in miR-338-5p overexpressing Capan-2 cells after 50 ng/ml EGF treatment for 48 h. **(C, D)** EGFR silencing reversed migration, invasion **(C)** and the related protein levels **(D)** in miR-338-5p silencing PANC-1 cells after 50 ng/ml EGF treatment for 48 h. *P < 0.05; **P < 0.01; ***P < 0.001.

Previous studies have demonstrated that ERK/MAPK signaling promotes EMT process and metastasis in many tumor types (9–11). To determine whether EGFR/ERK-mediated signaling participated in the inhibitory effect of miR-338-5p on EMT, we performed a rescue experiment by regulating miR-338-5p and EGFR in the same directions. First, western blot assays showed that the expression level of EGFR was significantly increased in cells infected with EGFR overexpression lentivirus and decreased in cells transfected with EGFR siRNA, compared with their respective control groups (**Figure S1**). Under EGF treatment, the effects of miR-338-5p on p-EGFR1068, E-cadherin, MMP9, and p-ERK were significantly reversed by the overexpression of EGFR in Capan-2 cells (**Figure 5B**). Furthermore, transwell assays showed that overexpression of EGFR also reversed the decreased Capan-2 migration and invasion induced by miR‐338-5p mimics (**Figure 5A**).

In addition, PANC-1 cell line was co-transfected with EGFR siRNA and inhibitor-338-5p. We found that EGFR knockdown significantly reversed the effects of miR-338-5p inhibitor on p-EGFR1068, E-cadherin, MMP9, and p-ERK (**Figure 5D**), and at the same time reversed the increased migration and invasion of PANC-1 induced by miR‐338-5p inhibitor (**Figure 5C**).

### miR‐338-5p Inhibited Liver Metastasis of Pancreatic Tumors *In Vivo*

**Figure 6:**
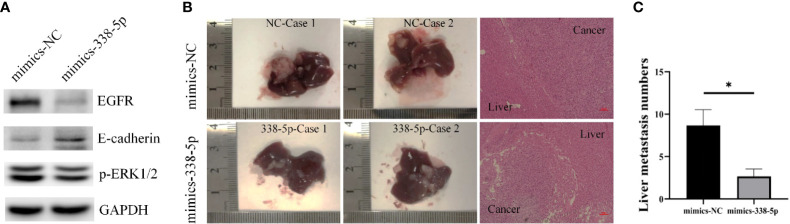
MiR-338-5p inhibited liver metastases *in vivo*. **(A)** the protein levels of EGFR, E-cadherin, and p-ERK in PANC02 cells transfected with mimics NC or miR-338-5p mimics detected by western blot. **(B)** Liver metastases and corresponding HE staining (100×) in mimics-NC and mimics-338-5p groups. **(C)** The statistical analysis of liver metastasis number between mimics-NC and mimics-338-5p groups. Bars indicate ± SEM. *P < 0.05 compared with the control.

By querying the miRbase software, the sequences of human and mouse miR-338-5p are proven to be completely identical (**Figure S1**), and there are potential binding sites for miR-338-5p in the 3’UTR of EGFR mRNA in mouse. PANC02 cells were used to construct the models of liver metastasis. First, we transfected PANC02 cells with the miR‐338-5p mimics or mimics NC, respectively. The transfection efficiency of miR-338-5p was detected by qRT‐PCR (**Figure S1**). By western blot analysis, PANC02 cell line showed minimal expression of E-cadherin. MiR-338-5p overexpression significantly downregulated EGFR, and p-ERK, but upregulated E-cadherin expression at protein level (**Figure 6A**). When the mice were sacrificed, the liver tissues were dissected and HE staining was performed to confirm liver metastasis. The liver metastases in mimics-338-5p group were significantly lower than that in mimics-NC groups (P = 0.04332) (**Figures 6B, C**).

## Discussion

EGFR belongs to the HER family of tyrosine kinase, which is widely distributed on the surface of mammalian cell membranes. EGFR plays an important role in the etiology and progression of many carcinomas, including PC. In pancreatic ductal carcinoma, EGFR is overexpressed in 30–89% of the cases (12). Valsecchi et al. found that EGFR was overexpressed in 30.4% of cases diagnosed with PCDA and these patients were found to always have lymph node metastasis (P = 0.038) and generally shorter survival rates (13). In our study, we found that the positive rate of EGFR in PC tissues was 72.5%, which was higher than that in adjacent normal tissues. In addition, according to the survival data of the TCGA database, the high expression of EGFR was related to the shorter survival time of patients and this was consistent with previous results.

miRNAs are small noncoding RNA molecules whose size ranges 19 to 24 nt and can interfere with gene expression through degradation of mRNA or inhibition of translational machinery (14). Study has shown that miR-338-5p could increase sensitivity of hepatoma cells to doxorubicin (15). This tumor suppressive function was also confirmed in glioma (16, 17), and esophageal squamous cell cancer (18, 19). But in colorectal cancer, miR-338-5p was identified as a potential diagnostic biomarker (20, 21), and promoted CRC progression (22, 23). However, its expression and function in pancreatic cancer have not been reported so far.

The results of our present study showed that miR-338-5p was downregulated in PC tissues than normal pancreatic tissues. Meanwhile, low expression of miR-338-5p was associated with lymph node metastasis and higher AJCC stage. Furthermore, the overexpression of miR-338-5p significantly suppressed the EGF-induced EMT, migration, and invasion of PANC-1 and Capan-2 cells. In contrast, miR-338-5p inhibitors enhanced migration, and invasion of PANC-1 and Capan-2 cells with EGF treatment. Additionally, EGFR was predicted to be a target protein of miR-338-5p by bioinformatic analysis, and a negative association of miR-338-5p and EGFR expression was observed. All the above results indicated that miR-338-5p may act as a potential tumor suppressor through post-transcriptional regulation of EGFR in pancreatic cancer.

EGF is considered to be one of the most significant ligands out of all the ligands that have the ability to bind to and activate EGFR (24). Activation of the EGFR kinase stimulates the following two signaling pathways: Ras/Raf/MEK/ERK and PI3K/Akt/mTOR (25). According to recent report, miR-338-5p inhibited the growth and invasion of trophoblast cells by targeting EFEMP1/Akt (26). However, in current study of PC Capan-2 and PANC-1 cells, we did not find the regulatory effect of miR-338-5p on the expression of p-Akt (S473) and mTOR protein. In fact, this is not surprising, because the same miRNA may have different mechanisms depending on the cellular environment.

Another study in colorectal cancer (CRC) demonstrated that miR-338-5p induced EMT by suppressing PIK3C3 expression and autophagy (27), which indicates that autophagy inhibits EMT in CRC. However, the protein level of ATG5 is positively related to the invasiveness of human pancreatic cancer, whereas the deletion of Atg5 inhibits tumor proliferation and metastasis in PDAC (28). It is worth noting that, autophagy has a double-edged sword effect in cancer (29, 30). The effect of autophagy on EMT appears controversial, which depends on cell type and the stage of cancer (31). Therefore, in pancreatic cancer, the effect of miR-338-5p on EMT by inhibiting PIK3C3 and autophagy is still unclear and needs further study.

Although miR-338-5p/EGFR axis has been shown to inhibit multidrug resistance and cell growth in hepatocellular carcinoma (15), the results of the present study indicate that miR-338-5p/EGFR axis inhibits the metastasis of pancreatic cancer partially dependent on EGF. With EGF stimulus, miR-338-5p targeted EGFR and downregulated EGFR, p-EGFR1068, p-EGFR1045, and p-ERK, subsequently increased E-cad protein expression in PC cells. In the absence of EGF stimulation, however, the regulatory effect of miR-338-5p on the downstream of EGFR was not very significant and this phenomenon was especially reflected in the weak changes of p-EGFR1068 and 1045. Finally, rescue experiments were carried out to verified above results. Here, we believe that the inhibition of EGFR/ERK signal by miR-338-5p is amplified by EGF stimulation.

In summary, we found that miR-338-5p inhibited the EMT process of PC cells by specially regulating EGF activated EGFR/ERK signaling. Although the KRAS proto-oncogene point mutation occurs in 90% of PC (32, 33), the unique activities of EGFR were demonstrated to promote cancer progression even when KRAS is mutated in PC cell lines (34–38). Considering that the robust functions of EGFR in PC, the novel identified miR-338-5p/EGFR/ERK axis may provide new insight into the underlying mechanism of PC progression, and the restoration of miR-338-5p could provide a therapeutic strategy for advanced PC.

## Data Availability Statement

The raw data supporting the conclusions of this article will be made available by the authors, without undue reservation.

## Ethics Statement

The studies involving human participants were reviewed and approved by the Ethics Committee of China Medical University. Written informed consent to participate in this study was provided by the participants’ legal guardian/next of kin. The animal study was reviewed and approved by the Ethics Committee of China Medical University. Written informed consent was obtained from the individual(s), and minor(s)’ legal guardian/next of kin, for the publication of any potentially identifiable images or data included in this article.

## Author Contributions

JS conceived and designed the experiments. JS and LC performed the experiments and analyzed the data. JS wrote the manuscript. MD revised and corrected the manuscript. All authors contributed to the article and approved the submitted version.

## Funding

This work was supported by the Chinese National Science Foundation (No. 81672835 to MD).

## Conflict of Interest

The authors declare that the research was conducted in the absence of any commercial or financial relationships that could be construed as a potential conflict of interest.

## Supplementary Material

Supplementary Figure 1 Supplementary material in the article. **(A)** The vector information of EGFR overexpression lentivirus. **(B, C)** The transfection efficiency of miR-338-5p was detected by qRT‐PCR. **(D)** The EGFR protein levels of Capan-2 and PANC-1 cells transfected with miR-338-5p mimics or inhibitor, compared with negative control (the histogram corresponding to Panels 2C, D). **(E)** With or without EGF treatment, the protein levels of p-mTOR and p-Akt in Capan-2 and PANC-1 cells transfected with mimics NC or miR-338-5p mimics detected by western blot. **(F)** The protein levels of EGFR Capan-2 and PANC-1 cells infected with EGFR overexpression lentivirus or transfected with EGFR siRNA. **(G)** The sequences of human and mouse miR-338-5p. Bars indicate ± SEM. *P < 0.05; **P < 0.01; ***P < 0.001; ns, no significance.
