# Supplementary figures and images for: MiR-338-5p Inhibits EGF-Induced EMT in Pancreatic Cancer Cells by Targeting EGFR/ERK Signaling
Source: Front Oncol. 2021 Apr 15;11:616481. doi: 10.3389/fonc.2021.616481 (PMC8082406; doi:10.3389/fonc.2021.616481)

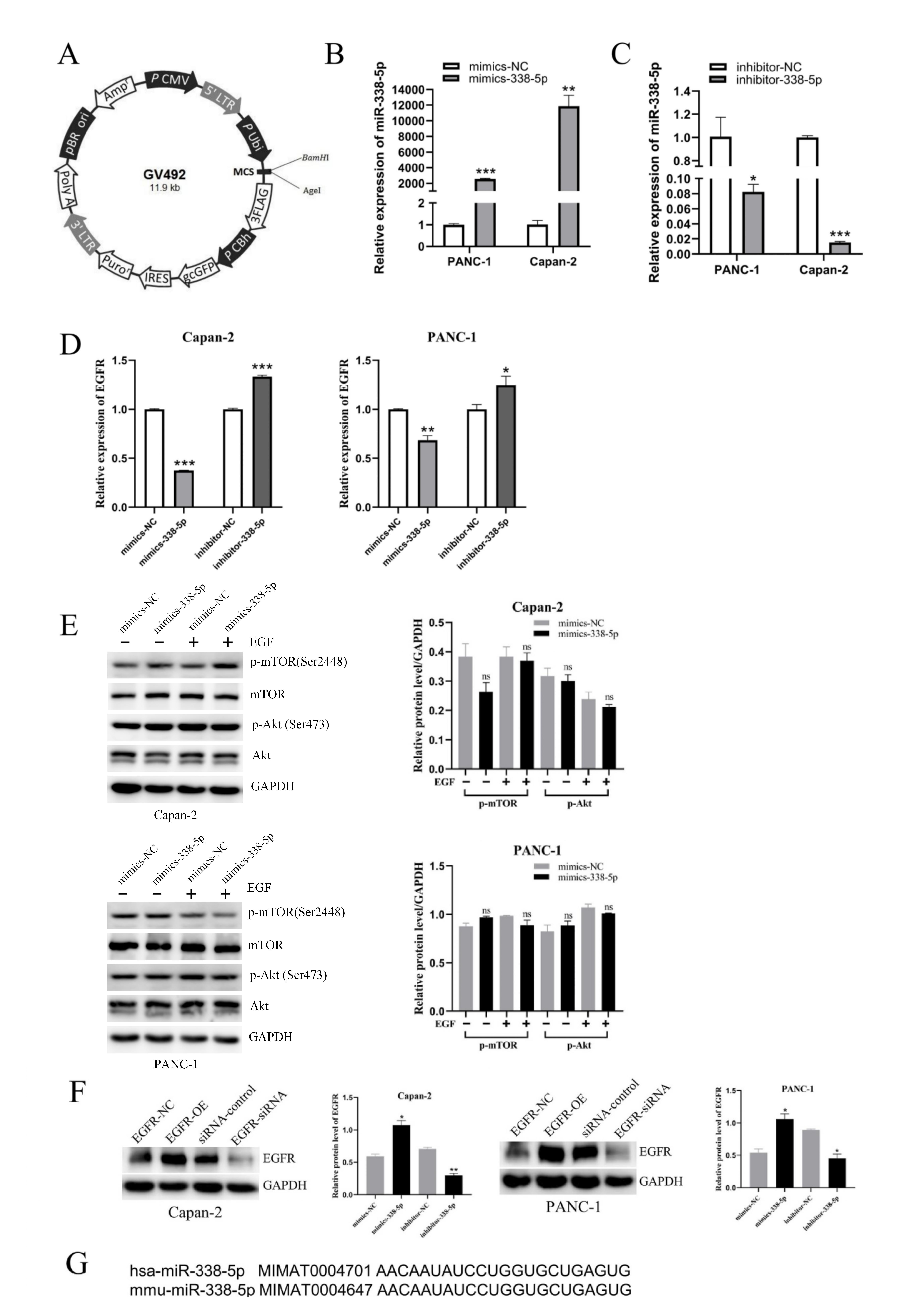

Supplement: Supplementary Figure 1 — Supplementary material in the article. (A) The vector information of EGFR overexpression lentivirus. (B, C) The transfection efficiency of miR-338-5p was detected by qRT‐PCR. (D) The EGFR protein levels of Capan-2 and PANC-1 cells transfected with miR-338-5p mimics or inhibitor, compared with negative control (the histogram corresponding to Panels 2C, D). (E) With or without EGF treatment, the protein levels of p-mTOR and p-Akt in Capan-2 and PANC-1 cells transfected with mimics NC or miR-338-5p mimics detected by western blot. (F) The protein levels of EGFR Capan-2 and PANC-1 cells infected with EGFR overexpression lentivirus or transfected with EGFR siRNA. (G) The sequences of human and mouse miR-338-5p. Bars indicate ± SEM. *P < 0.05; **P < 0.01; ***P < 0.001; ns, no significance. [file Image_1.jpeg]
