# Supplementary material for: MiR-338-5p Inhibits EGF-Induced EMT in Pancreatic Cancer Cells by Targeting EGFR/ERK Signaling
Source: Front Oncol. 2021 Apr 15;11:616481. doi: 10.3389/fonc.2021.616481 (PMC8082406; doi:10.3389/fonc.2021.616481)
Supplement: Supplementary file 2 [file DataSheet_1.docx]

The related insert sequences of EGFR overexpression lentivirus:

ATGCGACCCTCCGGGACGGCCGGGGCAGCGCTCCTGGCGCTGCTGGCTGCGCTCTGCCCGGCGAGTCGGGCTCTGGAGGAAAAGAAAGTTTGCCAAGGCACGAGTAACAAGCTCACGCAGTTGGGCACTTTTGAAGATCATTTTCTCAGCCTCCAGAGGATGTTCAATAACTGTGAGGTGGTCCTTGGGAATTTGGAAATTACCTATGTGCAGAGGAATTATGATCTTTCCTTCTTAAAGACCATCCAGGAGGTGGCTGGTTATGTCCTCATTGCCCTCAACACAGTGGAGCGAATTCCTTTGGAAAACCTGCAGATCATCAGAGGAAATATGTACTACGAAAATTCCTATGCCTTAGCAGTCTTATCTAACTATGATGCAAATAAAACCGGACTGAAGGAGCTGCCCATGAGAAATTTACAGGAAATCCTGCATGGCGCCGTGCGGTTCAGCAACAACCCTGCCCTGTGCAACGTGGAGAGCATCCAGTGGCGGGACATAGTCAGCAGTGACTTTCTCAGCAACATGTCGATGGACTTCCAGAACCACCTGGGCAGCTGCCAAAAGTGTGATCCAAGCTGTCCCAATGGGAGCTGCTGGGGTGCAGGAGAGGAGAACTGCCAGAAACTGACCAAAATCATCTGTGCCCAGCAGTGCTCCGGGCGCTGCCGTGGCAAGTCCCCCAGTGACTGCTGCCACAACCAGTGTGCTGCAGGCTGCACAGGCCCCCGGGAGAGCGACTGCCTGGTCTGCCGCAAATTCCGAGACGAAGCCACGTGCAAGGACACCTGCCCCCCACTCATGCTCTACAACCCCACCACGTACCAGATGGATGTGAACCCCGAGGGCAAATACAGCTTTGGTGCCACCTGCGTGAAGAAGTGTCCCCGTAATTATGTGGTGACAGATCACGGCTCGTGCGTCCGAGCCTGTGGGGCCGACAGCTATGAGATGGAGGAAGACGGCGTCCGCAAGTGTAAGAAGTGCGAAGGGCCTTGCCGCAAAGTGTGTAACGGAATAGGTATTGGTGAATTTAAAGACTCACTCTCCATAAATGCTACGAATATTAAACACTTCAAAAACTGCACCTCCATCAGTGGCGATCTCCACATCCTGCCGGTGGCATTTAGGGGTGACTCCTTCACACATACTCCTCCTCTGGATCCACAGGAACTGGATATTCTGAAAACCGTAAAGGAAATCACAGGGTTTTTGCTGATTCAGGCTTGGCCTGAAAACAGGACGGACCTCCATGCCTTTGAGAACCTAGAAATCATACGCGGCAGGACCAAGCAACATGGTCAGTTTTCTCTTGCAGTCGTCAGCCTGAACATAACATCCTTGGGATTACGCTCCCTCAAGGAGATAAGTGATGGAGATGTGATAATTTCAGGAAACAAAAATTTGTGCTATGCAAATACAATAAACTGGAAAAAACTGTTTGGGACCTCCGGTCAGAAAACCAAAATTATAAGCAACAGAGGTGAAAACAGCTGCAAGGCCACAGGCCAGGTCTGCCATGCCTTGTGCTCCCCCGAGGGCTGCTGGGGCCCGGAGCCCAGGGACTGCGTCTCTTGCCGGAATGTCAGCCGAGGCAGGGAATGCGTGGACAAGTGCAACCTTCTGGAGGGTGAGCCAAGGGAGTTTGTGGAGAACTCTGAGTGCATACAGTGCCACCCAGAGTGCCTGCCTCAGGCCATGAACATCACCTGCACAGGACGGGGACCAGACAACTGTATCCAGTGTGCCCACTACATTGACGGCCCCCACTGCGTCAAGACCTGCCCGGCAGGAGTCATGGGAGAAAACAACACCCTGGTCTGGAAGTACGCAGACGCCGGCCATGTGTGCCACCTGTGCCATCCAAACTGCACCTACGGATGCACTGGGCCAGGTCTTGAAGGCTGTCCAACGAATGGGCCTAAAATCCCGTCCATCGCCACTGGGATGGTGGGGGCCCTCCTCTTGCTGCTGGTGGTGGCCCTGGGGATCGGCCTCTTCATGCGAAGGCGCCACATCGTTCGGAAGCGCACGCTGCGGAGGCTGCTGCAGGAGAGGGAGCTTGTGGAGCCTCTTACACCCAGTGGAGAAGCTCCCAACCAAGCTCTCTTGAGGATCTTGAAGGAAACTGAATTCAAAAAGATCAAAGTGCTGGGCTCCGGTGCGTTCGGCACGGTGTATAAGGGACTCTGGATCCCAGAAGGTGAGAAAGTTAAAATTCCCGTCGCTATCAAGGAATTAAGAGAAGCAACATCTCCGAAAGCCAACAAGGAAATCCTCGATGAAGCCTACGTGATGGCCAGCGTGGACAACCCCCACGTGTGCCGCCTGCTGGGCATCTGCCTCACCTCCACCGTGCAACTCATCACGCAGCTCATGCCCTTCGGCTGCCTCCTGGACTATGTCCGGGAACACAAAGACAATATTGGCTCCCAGTACCTGCTCAACTGGTGTGTGCAGATCGCAAAGGGCATGAACTACTTGGAGGACCGTCGCTTGGTGCACCGCGACCTGGCGGCCAGGAACGTACTGGTGAAAACACCGCAGCATGTCAAGATCACAGATTTTGGGCTGGCCAAACTGCTGGGTGCGGAAGAGAAAGAATACCATGCAGAAGGAGGCAAAGTGCCTATCAAGTGGATGGCATTGGAATCAATTCTACACAGAATCTATACCCACCAGAGTGATGTCTGGAGCTACGGGGTGACCGTTTGGGAGTTGATGACCTTTGGATCCAAGCCATATGACGGAATCCCTGCCAGCGAGATCTCCTCCATCCTGGAGAAAGGAGAACGCCTCCCTCAGCCACCCATATGTACCATCGATGTCTACATGATCATGGTCAAGTGCTGGATGATAGACGCAGATAGTCGCCCAAAGTTCCGTGAGTTGATCATCGAATTCTCCAAAATGGCCCGAGACCCCCAGCGCTACCTTGTCATTCAGGGGGATGAAAGAATGCATTTGCCAAGTCCTACAGACTCCAACTTCTACCGTGCCCTGATGGATGAAGAAGACATGGACGACGTGGTGGATGCCGACGAGTACCTCATCCCACAGCAGGGCTTCTTCAGCAGCCCCTCCACGTCACGGACTCCCCTCCTGAGCTCTCTGAGTGCAACCAGCAACAATTCCACCGTGGCTTGCATTGATAGAAATGGGCTGCAAAGCTGTCCCATCAAGGAAGACAGCTTCTTGCAGCGATACAGCTCAGACCCCACAGGCGCCTTGACTGAGGACAGCATAGACGACACCTTCCTCCCAGTGCCTGAATACATAAACCAGTCCGTTCCCAAAAGGCCCGCTGGCTCTGTGCAGAATCCTGTCTATCACAATCAGCCTCTGAACCCCGCGCCCAGCAGAGACCCACACTACCAGGACCCCCACAGCACTGCAGTGGGCAACCCCGAGTATCTCAACACTGTCCAGCCCACCTGTGTCAACAGCACATTCGACAGCCCTGCCCACTGGGCCCAGAAAGGCAGCCACCAAATTAGCCTGGACAACCCTGACTACCAGCAGGACTTCTTTCCCAAGGAAGCCAAGCCAAATGGCATCTTTAAGGGCTCCACAGCTGAAAATGCAGAATACCTAAGGGTCGCGCCACAAAGCAGTGAATTTATTGGAGCA
